# Isolated Testicular Metastasis From Prostate Cancer: A Rare Presentation 15 Years Posttreatment

**DOI:** 10.7759/cureus.78571

**Published:** 2025-02-05

**Authors:** Tauqir Aslam Waraich, Syed Yousaf Khalid, Asadullah Aslam, Claudio Lizarralde, Syed Jaffry

**Affiliations:** 1 Department of Urology, Letterkenny University Hospital, Letterkenny, IRL; 2 Department of Urology, University Hospital Galway, Galway, IRL; 3 Department of Cardiothoracic Surgery, St. James's Hospital, Dublin, IRL; 4 Department of Urology, Kingsbridge Healthcare Group, Belfast, IRL; 5 Department of Histopathology, Galway Clinic, Galway, IRL; 6 Department of Urology, Galway Clinic, Galway, IRL

**Keywords:** androgen deprivation therapy, prostate-specific antigen, prostatic adenocarcinoma, testes, testicular metastasis

## Abstract

Testicular metastases from prostate cancer (PCa) are exceedingly rare and are typically accompanied by other metastatic sites. We present the case of an 82-year-old male patient who developed isolated testicular metastasis 15 years after undergoing radiotherapy and androgen deprivation therapy (ADT) for localized PCa. The patient presented with a palpable left testicular mass, which was confirmed to be a metastatic prostate adenocarcinoma by histopathological and immunohistochemical analyses. Orchiectomy was performed for both diagnostic and therapeutic purposes, resulting in a significant decline in serum PSA levels. The patient continued ADT with regular monitoring, and no further metastases were detected. This case highlights the importance of long-term surveillance and advanced diagnostic techniques for detecting the rare metastatic presentations of PCa. Although isolated testicular metastases may have a better prognosis than other metastatic patterns, optimal postorchiectomy management remains uncertain, underscoring the need for further research and a multidisciplinary approach.

## Introduction

Prostate cancer (PCa) is the most frequently diagnosed cancer in men in 112 of 185 countries worldwide. It exhibits varying patterns of incidence across different regions, ranking second (37.5 per 100,000) behind lung cancer in higher Human Development Index (HDI) countries but taking the lead in lower HDI countries with 11.3 per 100,000, surpassing lung cancer at 10.3 per 100,000 [1]. Projections based on global demographic trends and increasing life expectancy estimate that the annual number of new PCa cases will rise from 1.4 million in 2020 to 2.9 million by 2040 [2]. PCa has a 97% five-year cancer-specific survival rate when diagnosed and treated at localized stages compared to only 30% in cases of metastatic disease [3]. PCa may spread through local invasion (primarily to the bladder and seminal vesicles, with rare involvement of the urethra and rectum), lymphatic pathways (initially affecting pelvic nodes, followed by para-aortic and inguinal nodes), or hematogenous routes. The most common metastatic sites are the bone (90%), lungs (46%), liver (25%), pleura (21%), and adrenal glands (13%) [4]. Testicular metastases from PCa are exceedingly rare, occurring in only 0.5% of cases identified through autopsies [4]. Here, we present a rare case of isolated metastatic prostate adenocarcinoma manifesting as a palpable testicular mass 15 years after initial diagnosis and treatment.

## Case presentation

An 82-year-old male patient presented to the outpatient department with discomfort caused by a palpable mass in his left testis that had persisted for one month. The mass was insidious at onset and gradually increased in size. His medical history included a diagnosis of PCa in 2006, for which he underwent radiotherapy (RT) and two years of adjuvant androgen deprivation therapy (ADT). At the time of diagnosis, metastatic evaluation, including a bone scan and computed tomography (CT) of the abdomen and pelvis, revealed no evidence of metastasis. The patient’s prostate-specific antigen (PSA) level was 21 ng/mL (reference range: 0-4 ng/mL), and transrectal ultrasound-guided biopsy revealed a Gleason score of 3+4 adenocarcinoma with a clinical stage of cT2b.

**Figure 1:**
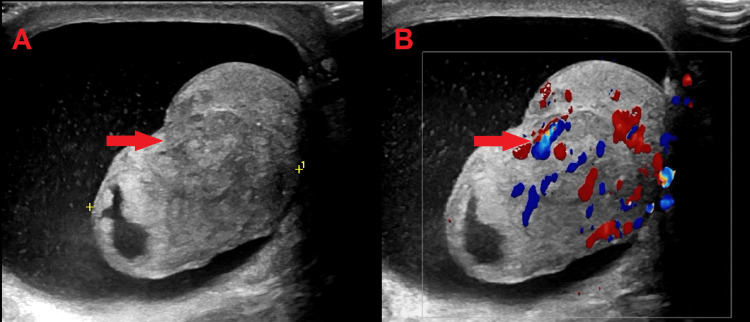
Ultrasound and color Doppler imaging of left testicular lesion. (A) Grayscale image depicting a 2.4-cm well-encapsulated, hyperechoic intratesticular mass (red arrow) highly suspicious for malignancy. (B) Color Doppler image showing significant vascular flow within the lesion (red arrow)

Following RT and ADT, the patient’s PSA levels reached a nadir of 0.02-0.03 ng/mL and remained stable for many years. However, in 2021, PSA levels rose, reaching 5.2 ng/mL (reference range: 0-2.03 ng/mL). Upon presentation, physical examination revealed a firm, nontender mass in the left testicle with no associated inguinal lymphadenopathy. Laboratory test results, including complete blood count, renal function, liver function, and tumor markers for testicular tumors (lactate dehydrogenase, alpha-fetoprotein, and beta-human chorionic gonadotropin, HCG), were all within normal limits. Scrotal ultrasonography (Figure 1) demonstrated a left intratesticular solid mass with a slightly heterogeneous hyperechoic appearance. Repeat CT and bone scans revealed no evidence of metastatic recurrence.

**Figure 2:**
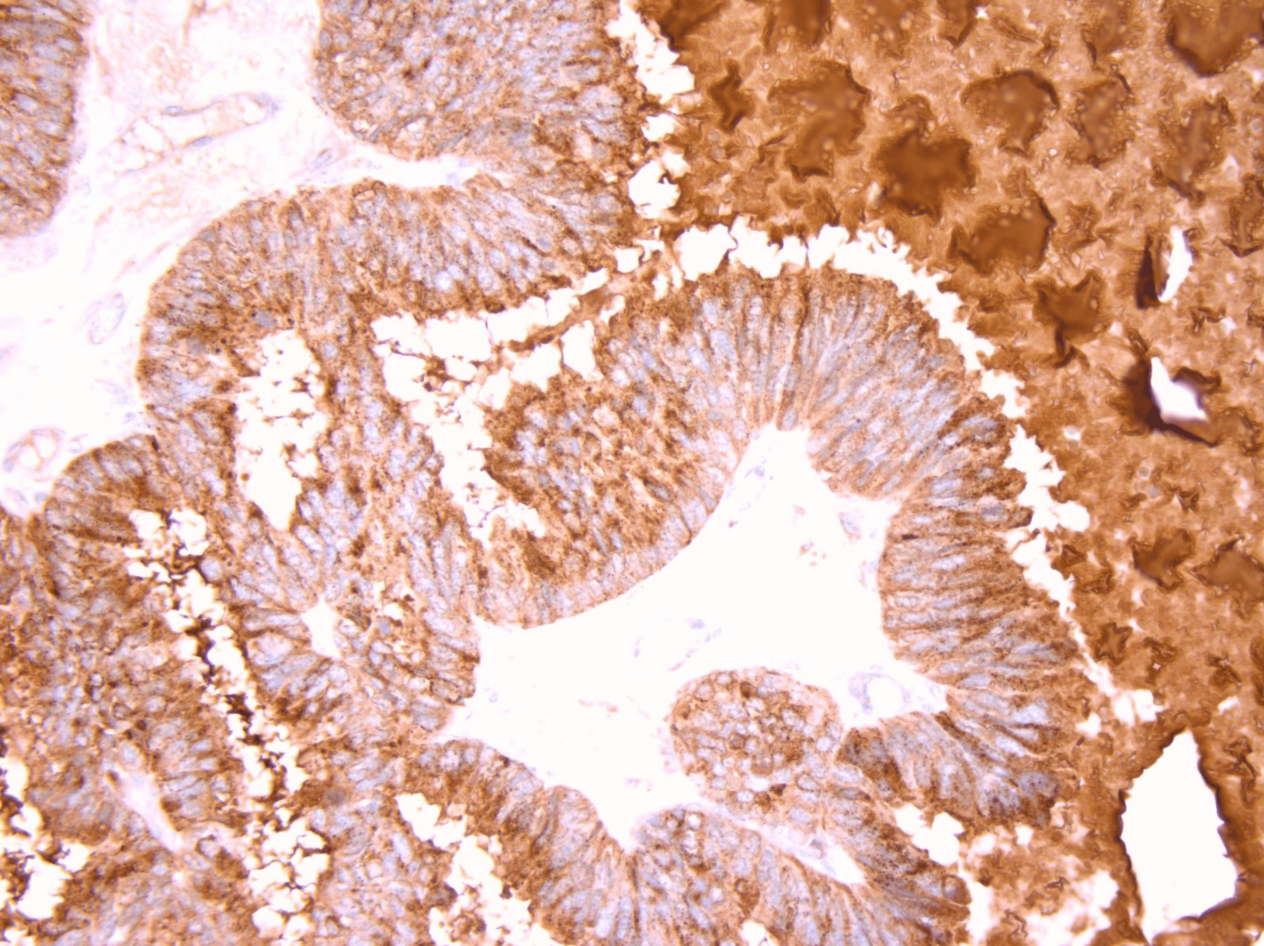
Immunohistochemical staining demonstrating metastatic ductal prostate adenocarcinoma in testicular tissue

In February 2021, the patient underwent left inguinal orchiectomy, and ADT was initiated. Histopathological examination of the 2.5 × 3.1 cm testicular mass revealed a metastatic adenocarcinoma with a complex intraductal papillary pattern of tall columnar cells (Figure 2). The adjacent testis exhibited atrophy with prominent interstitial fibrosis, but no intratubular germ cell neoplasia was identified, and the epididymis was tumor-free. Immunohistochemical analysis confirmed the diagnosis of metastatic prostate adenocarcinoma of the ductal type, with positive staining for cytokeratin AE1/AE3, epithelial membrane antigen, PSA, and prostatic intraepithelial neoplasia marker (PIN), and negative staining for cluster of differentiation 30, cluster of differentiation 117, vimentin, octamer-binding transcription factor 4 (OCT-4), placental alkaline phosphatase (PLAP), and HCG.

The patient’s serum PSA level decreased to 0.03 ng/mL at follow-up. The patient continued to receive ADT with regular monitoring of PSA levels.

## Discussion

Testicular metastases from PCa are uncommon, occurring in only 0.5% of cases and are typically identified incidentally during autopsies or therapeutic orchiectomies in patients with advanced PCa [4,5]. The rarity of testicular metastasis can be attributed to several factors. One explanation is the lower temperature of the scrotum due to its extracorporeal position, which is believed to inhibit tumor growth. Another hypothesis suggests that Sertoli cells may play a crucial role by forming a blood-testis barrier, which limits the hematogenous spread of tumor cells [6].

Several mechanisms have been proposed to explain the metastasis of PCa to the testes, including arterial embolism, migration through the vas deferens lumen, retrograde venous extension, and retrograde lymphatic spread [4].

Testicular metastasis typically presents as a painless testicular mass [7-9], as observed in this patient. Imaging modalities, such as scrotal ultrasonography, and advanced techniques, such as prostate-specific membrane antigen positron-emission tomography (PSMA PET/CT), play crucial roles in identifying rare metastatic sites [9-11]. Histopathological evaluation is essential for definitive diagnosis. In our case, immunohistochemical analysis was critical, with the tumor expressing prostate-specific markers such as PSA and PIN and negative for germ cell markers (e.g., OCT-4, PLAP, and HCG), consistent with previous reports [12].

The survival rate following a diagnosis of testicular metastasis from primary PCa is generally less than one year [12-14]. However, the prognosis of patients with isolated testicular metastasis remains uncertain. Some authors suggest that this rare form of metastasis may have a lower potential for dissemination and a relatively better prognosis than other metastatic patterns [5,15].

The management of testicular metastasis in PCa typically involves orchiectomy, which serves diagnostic and therapeutic purposes. In the present case, left inguinal orchiectomy resulted in a dramatic decline in serum PSA levels, reflecting effective disease control. The optimal therapeutic approach following orchiectomy, in the absence of additional metastases, remains a matter of debate. Although a solitary secondary localization to the testis from PCa may have limited potential for further spread, it could also represent an intermediate stage in the progression toward systemic dissemination. It is unclear whether strict monitoring alone is sufficient after achieving clinical and biochemical remission or if “adjuvant” treatment, as recommended by Kwon et al. [16], is necessary to ensure better long-term outcomes.

Although rare, isolated testicular metastases from PCa are highlighted by a few case reports and reviews. Bonetta et al. described a patient who developed a solitary testicular metastasis years after undergoing radical prostatectomy, highlighting the critical need for extended surveillance in high-risk cases [5]. DiMarco et al. described a case in which a solitary testicular lesion was detected through physical examination despite the patient’s low PSA levels, emphasizing the importance of comprehensive clinical assessments [15]. McCann et al. presented a similar instance of a solitary testicular mass appearing years after initial treatment, with no concurrent systemic disease, illustrating the unpredictable progression of such metastases [12]. Herrera Ortiz et al. demonstrated the utility of advanced imaging modalities, such as PSMA PET-CT, in detecting these rare metastases, which might otherwise remain unnoticed during standard follow-ups [10]. Gupta et al. further highlighted the diagnostic challenges posed by the histopathological and immunohistochemical similarities between testicular metastases and primary PCa, emphasizing the importance of a multidisciplinary approach to diagnosis and management [11]. These reports emphasize the clinical complexities and diagnostic challenges of isolated testicular metastases and advocate for vigilant long-term monitoring of patients with PCa.

## Conclusions

This case highlights the rarity of isolated testicular metastases in PCa and underscores the need for vigilance and thorough evaluation. The 15-year interval from the initial diagnosis to metastasis demonstrates an unpredictable progression of the disease. Advanced imaging and immunohistochemical analysis are crucial for accurate diagnosis, while orchiectomy serves diagnostic and therapeutic purposes, with ADT aiding sustained remission. Optimal postorchiectomy management remains uncertain, highlighting the need for further research. This case emphasizes the importance of long-term surveillance and a multidisciplinary approach to managing patients with PCa.
